# NK Cells in HIV Disease

**DOI:** 10.1007/s11904-016-0310-3

**Published:** 2016-03-21

**Authors:** Eileen Scully, Galit Alter

**Affiliations:** Ragon Institute of Massachusetts General Hospital, Massachusetts Institute of Technology and Harvard, 400 Technology Square, Cambridge, MA 02139 USA; Division of Infectious Disease, Department of Medicine, Brigham and Women’s Hospital, Boston, MA 02130 USA

**Keywords:** HIV, Innate immunity, NK cell, Antibody-dependent cellular cytotoxicity, NK cell memory, Natural killer (NK), Viral immunity, HIV pathogenesis, Review

## Abstract

Natural killer (NK) cells play a critical role in viral immunity. In the setting of HIV infection, epidemiologic and functional evidence support a role for NK cells in both protection from new infection and in viral control. Specifically, NK cells directly mediate immune pressure leading to virus evolution, and NK cell receptor genotypic profiles, clonal repertoires, and functional capacity have all been implicated in virus containment. In addition, indirect NK cell-mediated antibody-dependent cellular cytotoxicity has been linked to vaccine-induced protective immunity against HIV infection. With recent advances in our understanding of NK cell deficiency, development, memory-like responses, and editing of the adaptive immune system, the opportunities to direct and exploit NK cell antiviral immunity to target HIV have exponentially grown. In this review, we seek to highlight the intersections between discoveries in basic NK cell biology and the challenges of HIV chronic infection, vaccine development, and cure/eradication strategies.

## Introduction

**Fig. 1:**
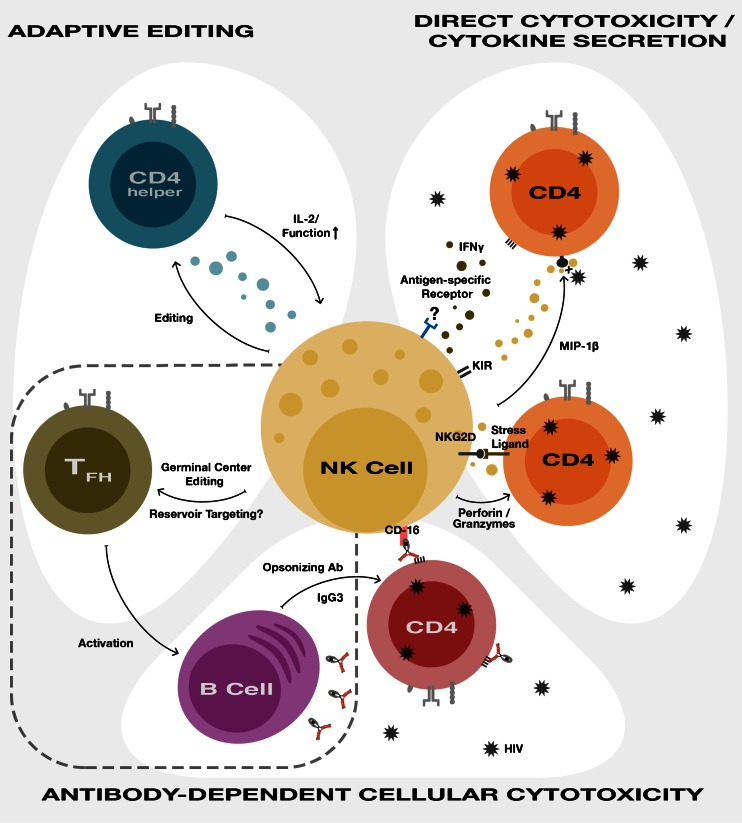
Multiple facets of NK cell biology can be harnessed towards the goal of HIV prevention and eradication. (*Right panel*) NK cells can directly recognize HIV-infected targets through expression of stress ligands on infected cells or potentially through antigen-specific responses as has been demonstrated in the SIV infection in rhesus macaques. This recognition may lead to degranulation and target cell cytolysis. In addition, secretion of chemokines such as MIP-1β can block new rounds of infection and effectors such as IFNγ can activate antiviral programs. (*Bottom panel*) NK cells can also be indirectly recruited to HIV-infected cells through CD16 engagement of the Fc receptor of antibodies bound to HIV epitopes. Optimization of these antibodies through subtypes with multiple coordinated activities and glycan patterns that direct maximal responses can enhance NK effector function. (*Left panel*) NK cell cross-talk with the adaptive immune system also affords opportunities for modulating responses. NK cells edit T follicular helper (T_FH_) cells altering germinal center formation and antibody development through cross-talk with B cells. T_FH_ cells are also a reservoir of HIV infection, and could potentially be targeted by NK cells in early infection or during curative interventions. NK cell editing of CD4^+^ T cell responses also contributes to immune exhaustion in chronic viral infection models, and limits immunopathology in acute infections that are cleared. There is the potential for bidirectional effects with NK cell limiting or enhancing CD4 function, and CD4 T cell cytokine production supporting improved NK cell effector capacity. In sum, there are multiple avenues through which NK cell function can be leveraged to optimize immune responses to HIV

Natural killer (NK) cells occupy a unique niche in the immune response, bridging the innate and adaptive immune systems. They are capable of recognizing generic signals of stress, transformation, or infection with immediate effector function. NK cells are the critical antiviral effectors of the innate immune system, and natural deficiencies are associated with susceptibility to viral infections [1, 2]. However, there is still a nascent understanding of their pleiotropic functions as direct effectors, but also as editors of adaptive immunity in both infection and vaccination [3]. Recent work has also highlighted the adaptive features of NK cells that can confer a memory-like phenotype in the setting of infection [4]. NK cells have the potential to directly respond to viruses, to develop memory-like responses after initial pathogen encounter or vaccination, and to shape the adaptive immune response (Fig. 1).

As the understanding of NK cell biology grows, there are promising avenues to address several current challenges in HIV: development of an effective vaccine, control of residual inflammation in treated disease, and emerging strategies for eradication or cure. NK cell deficiency syndromes have revealed specific developmental programs that shape the mature NK cell population and can broadly modulate responses [5]. The HIV vaccine literature has identified the critical importance of antibody-dependent cellular cytotoxicicty (ADCC), mediated predominantly by NK cells, via non-neutralizing antibodies as a pathway to protective vaccine responses [6]. Importantly, these ADCC responses are associated with specific immunoglobulin subclasses [7] and glycan modifications that directly impact the efficiency of NK cell responses [8, 9]. This affords multiple avenues for optimization of antibodies to maximize NK cell function for preventative and therapeutic vaccination [10].

The profile of NK cell responsiveness beyond CD16-mediated antibody recognition also points to anti-HIV function. High dimensional characterization of the combinatorial diversity of NK receptor expression offers new insights into the phenotypic features of protective NK cell responses and how they may be directed [11, 12••]. Building on prior work showing antigen specificity in mouse models, recent studies have demonstrated NK cell memory responses to simian immunodeficiency virus (SIV) antigens in nonhuman primates, suggesting a pathway to induce antigen-driven memory in humans [13••]. In line with recent findings in the myeloid compartment delineating the phenomena of innate training and tolerance [14], unique epigenetic signatures in NK cells primed by cytomegalovirus (CMV) infection demonstrate how NK cells can be programmed by an initial pathogen (or potentially vaccine) exposure [15••, 16••]. Taken together, this work suggests infection can shape the NK cell repertoire and alter subsequent responses; a detailed understanding of this functional plasticity will inform efforts to develop preventative and therapeutic vaccines against HIV.

In this review, we discuss the role of NK cells in the immune response to HIV in the context of recent advances in NK cell biology, highlighting opportunities to harness these innate cytolytic effector cells for the rapid containment and clearance of HIV.

### NK Cells in HIV Infection

#### The Role of NK Cells in the Immune Response to HIV Infection

Population-level genetic associations between NK cell receptor expression and HIV-1 outcomes and evolution reveal the impact of NK cells on HIV-1 disease progression. The germline encoded inhibitory receptors including the killer immunoglobulin-like receptors (KIRs) heavily influence NK cell activation which is governed by the integration of activating and inhibitory signals. Interactions between KIRs and their cognate HLA ligands set a threshold for NK activity and have been shown to critically influence the course of viral infection, associating with resolution of hepatitis C infection [17]. In HIV, HLA/KIR combinations have been associated with the pace of disease progression [18, 19] and protection against disease acquisition [20, 21]. The mechanisms conferring this protection may include both NK cell education/licensing through inhibitory receptor ligation [22] and the direct interaction of KIRs with HIV-1-derived peptide motifs presented on HLA molecules. The latter is supported by virus evolution in the presence of specific KIRs; footprints in the HIV genome associate with KIR genotype. Specific viral variant/KIR combinations associate with differences in NK cell viral inhibition in vitro [23] and HLA/KIR combinations confer differences in HIV control [24]. The HLA/KIR interactions directed by specific HIV-derived peptides are further linked to measures of NK cell function in vitro and patterns of viral escape in population studies [25, 26]. These data suggest that NK cell activation threshold, determined in part by the genetic array of inhibitory receptors, and the virus-derived peptides available for presentation on host HLA cooperate to define the protective efficacy of NK cell responses. The latter could be exploited to harness function; computation approaches may be able to predict peptides that modulate NK function based on host HLA/KIR patterns to individualize vaccine design.

These data demonstrate direct interactions between NK cells and HIV peptides; however, it is not known whether these signals govern in vivo effector function and which of the overlapping recognition pathways is sufficient for an effective response. Although counterintuitive, focusing on immune responses that HIV has evolved to evade may point to highly effective means of NK activation. HIV-mediated downregulation of HLA molecules, which shields infected cells from killing by CD8^+^ T cells [27], theoretically offers the “missing self” trigger for NK cells. However, this is limited by HIV immune evasion; the downregulation of HLA A and B by Nef is coupled to preservation of HLA C and E, maintaining self signals, and preventing NK activation [28]. Likewise, HIV infection directly triggers the upregulation of stress ligands for cytotoxicity receptors including NKG2D [29, 30]. Again, HIV is able to limit expression of these ligands and other coactivating signals such as NTBA-4 through accessory proteins [29, 31]. While the precise contribution of each activation pathway in natural infection is difficult to prove, from the perspective of prevention and cure, each could be leveraged to optimize responses. Small molecule inhibitors disabling immune evasion through viral accessory proteins may enhance native or vaccine-induced immune recognition.

ADCC is a potent means of NK cell control of HIV-1 infection. NK cells express the FcγRIIIA receptor (CD16) that binds the constant (Fc) domain of IgG antibodies. CD16 engagement is a strong activator of NK cell function, and allows antigen-specific recruitment of NK responses. Importantly, ADCC activity was associated with the modest protective efficacy in the RV144 HIV vaccine trial [6] and has been implicated in phenotypes of viral control [32, 33]. Responses characterized by coordinated antibody function, including NK mediated ADCC and NK cytokine secretion/degranulation associated with viral controller phenotypes [34]. ADCC efficiency is linked to specific antibody features including subclass and glycosylation that can be modified to enhance NK cell recruitment and activation. NK cell maturation and education can further enhance HIV-specific ADCC activity [35, 36]. Of note, as with other effector pathways, ADCC is also limited by viral evasion: the viral accessory protein Vpu antagonizes the antiviral factor tetherin, altering the release of virus aggregates and disabling ADCC mediated viral recognition [37–41]. A small molecule inhibitor of vpu’s interaction with tetherin has recently been described suggesting an additional target for intervention [42]. An optimized approach would prime NK cells, induce antibodies with glycan and subclass features maximized for ADCC and could potentially be coupled with pharmacologic blockade of viral immune evasion to further enhance antiviral function.

The collective data highlight the importance of NK cells in HIV disease. There are multiple pathways for target elimination, all balanced by mechanisms of viral evasion that define kinetic windows of maximal response and opportunities for enhancement. In addition to these specific interactions, NK cell recognition of generic signals of stress and infection induced early in HIV infection is an important area for exploration. Optimized NK cell function can be achieved through directly augmenting NK cell function with priming and subset expansion or inhibitory receptor blockade, indirectly through optimization of antibodies to recruit NK cells, and potentially also through blockade of viral accessory proteins to limit immune evasion.

#### The Consequences of HIV Infection for NK Cell Distribution and Function

Chronic HIV-1 infection alters the population distribution and functional capacity of NK cells. Numerous studies have described these changes in the setting of HIV infection and have at times produced diverging and contradictory results. This lack of clarity is likely related to multiple factors: the dependence of NK cell function on the associated HLA genotype has not always been fully considered, many studies are cross sectional in nature and may not have adequately controlled for confounders between groups including CMV serostatus, age, and gender, and finally the diversity of NK cell phenotypes is not fully captured in a limited examination of markers [11, 12••].

NK cells are divided into subsets based on their expression of CD56 and CD16; these subsets are thought to represent stages in NK cell differentiation, although the precise relationship has not been fully defined. Based on immunodeficiency syndromes, both CD56^bright^ and CD56^dim^ populations contribute to antiviral immunity. CD56^bright^CD16^−^ cells are a minor population in healthy individuals with relatively limited cytotoxic capacity but strong production of cytokines. CD56^dim^ NK cells are the majority of the circulating population with less proliferative potential, increased cytotoxic capacity, and progressive acquisition of KIRs and markers including CD57 during differentiation. A third minor population is the CD56^neg^ NK cells that are CD16^+^; these cells have been difficult to characterize with their limited expression of lineage markers. NK cells express a broad complement of additional receptors including the predominantly inhibitory KIRs; the natural cytotoxicity receptors (NCRs) including NKp30, NKp44, and NKp46; the C-type lectin receptors including NKG2D, NKG2C, and NKG2A; signaling lymphocyte activation (SLAM) family receptors as well as the Fcγ receptor CD16 [43]. NK cell degranulation/cytotoxicity and cytokine secretion are governed by the overall balance of activating and inhibitory signals, with an important role for education through inhibitory signaling during development [44]. The roles of each subset in HIV-1 infection are still being defined.

During primary HIV infection, initiation of antiretroviral therapy was associated with reciprocal variation in the CD16^+^CD56^dim^ and CD56^high^ NK cells, with the latter, more immature population, increasing after therapy [45]. Initiation of therapy also changes immunoregulatory markers; modulation of galectin 9 and T cell immunoglobulin and mucin-domain containing molecule-3 (TIM-3) in early infection leads to enhanced NK cell activity, but in chronic HIV the interaction may contribute to NK cell dysfunction [46]. Dysregulated TIM-3 expression on NK cells is linked to impaired CD4 recovery after treatment, suggesting that NK cell dysfunction may contribute to a poor immunologic reconstitution in some individuals [47]. CD56^neg^CD16^+^ cells with reduced ADCC activity also accumulate during chronic infection, a defect that is significantly restored with viral suppression [48, 49]. Counterbalancing this restoration of NK cell ADCC is the decline in antibody conferred activity after initiation of antiretroviral therapy (ART) [50]. Thus, to fully exploit the potential for NK cell-mediated function in cure/eradication strategies through ADCC, restoration of subset distribution is an essential step, beyond optimizing antibody subclass, glycan structure and titer through vaccination or administration of a monoclonal.

In vitro studies have sought to dissect receptor patterns that lead to NK cell control of HIV infection. When exposed to infected CD4^+^ T cells, autologous NK cells expressing NKG2A responded with CD107a (a marker of degranulation), IFNγ, and CCL4 at the highest frequencies [51]. Profiling the NKG2A receptor expression in an individual may offer an index of their likelihood to have an effective NK cell response to a therapeutic intervention. In the setting of significant inflammation in a cohort in Uganda with HIV subtype D infection, impaired effector function (IFNγ production) and loss of a highly activated subset of NK cells has been described [52]. Further evidence of dysfunction is suggested by studies in the SIV nonhuman primate model demonstrating anergic NK cell accumulation in lymph nodes in chronic infection [53]. Research to redirect or rescue function will be informed by the more complete understanding of the pathology of HIV. An alternative window to identify highly effective activation pathways may be through the enhanced effector capability seen in NK cells from individuals with spontaneous viral control. One recent example is the recent report of a potential role for NK cells in the long-term suppression of HIV-1 in the VISCONTI cohort of post-treatment controllers (Scott-Algara, et al., Abstract 15,CROI 2015, Seattle, WA).

In support of the concept that NK cells can be directed, methods of bolstering NK cell functional capacity in HIV infection have been described. A therapeutic HIV vaccine that restored CD4^+^ T cell IL-2 production led to enhanced production of IFNγ by NK cells [54]. Secretion of IFNα by other cells drives NK cell activation and lysis of HIV-infected CD4^+^ T cells through NKp46- and NKG2D-mediated signaling [55]. The cross-talk between adaptive and NK cell responses is a critical means for optimizing NK cell function. Coordinating the recruitment and activity of multiple cell types, particularly in the setting of an inflamed and anergic immune system, may be the key to promoting more effective NK cell control of HIV.

### NK Cell Development

#### Genetic Determinants of NK Cell Deficiency Syndromes

Naturally occurring NK cell deficiency syndromes offer important insights into the biology of these cytolytic effectors. Deficiency syndromes are broadly divided into classical NK cell deficiency (CNKD) with numeric and functional deficits, and functional NK cell deficiency (FNKD) in which cells are present but poorly functional (reviewed in [5, 56]). Genetic defects associated with both types of syndrome have been identified. In CNKD1, mutations in the transcription factor *GATA2* confer deficits in peripheral NK cell numbers and function, with marked susceptibility to viral infections (varicella zoster virus, herpes simplex virus, cytomegalovirus, human papilloma virus) and mycobacterial infections. These patients specifically lack of the CD56^bright^ subset and show globally impaired expression of NKG2D [57]. A distinct syndrome, CNKD2, is linked to a mutation in *MCM4*, with a converse phenotype of absence of the CD56^dim^ population with clinical findings of recurrent viral infections and EBV-driven lymphoproliferative disorders [58]. The deficit in opposite subsets in these two syndromes is striking given their proposed developmental relationship and the susceptibility to viral infections seen in both patient groups. In a separate patient cohort with marked susceptibility to herpesvirus infections and a form of FNKD, studies identified a mutation in the membrane distal domain of CD16. This mutation abrogated an interaction between CD16 and CD2, preventing activation of spontaneous cytotoxicity responses despite preserved ADCC [59]. The efficiency of ADCC responses is also associated with control of herpes simplex virus (HSV). Higher affinity allotypes of CD16 and IgG are linked to control of HSV (versus recurrent outbreaks) and are associated with enhanced ADCC in vitro [60, 61]. Multiple additional genetic loci have been linked to deficiencies in NK cell number and function in combined immune deficiency syndromes [62].

Taken together, these findings highlight key functional and developmental aspects of NK cell biology. First, both the CD56^bright^ and CD56^dim^ populations have significant roles in control of virus infections. Second, expression of CD16 has dual significance, promoting cytotoxic activity triggered by CD2 ligation as well as mediating ADCC. Third, the efficiency of the ADCC response is associated with successful control of herpes virus, reinforcing the role of ADCC in phenotypes of elite control in HIV. These observations may inform some of the disruption seen with shifts in the CD56 expression with chronic HIV infection, and supports the pursuit of both direct antiviral and ADCC strategies in HIV eradication and cure efforts.

#### Homeostasis and Maturation Signals

Given the defects seen in chronic HIV infection, understanding key differentiation signals in NK cell biology may point to therapeutic strategies to shape or direct their responses. IL-15 is a master regulator of NK cell maturation, survival, and functional competence. IL-15 preferentially expands cytotoxic NK subsets, with less proliferative stimulus for regulatory T cells than IL-2, shifting the net balance towards activation [63]. Recent human clinical trials in patients with metastatic cancer have demonstrated proliferation and activation of NK cells after administration of recombinant human IL-15 [64••]. In this exploratory study, no objective remissions were achieved; however, there was preliminary evidence of immune activity seen in the clearance of lung lesions in two of the subjects [64••]. This particular approach is relevant to HIV cure strategies; enhancing innate surveillance by NK cells in concert with latent virus reactivation can circumvent limitations of traditional adaptive responses to HIV. Ongoing efforts to optimize delivery methods (e.g., with heterodimeric formats of the cytokine and a receptor subunit [65]) and synergistic combinations of cytokine with antibody or effector CTLs are active areas of investigation (Euler, Jones, and Alter, in preparation). In a humanized mouse model of HIV infection, use of an IL-15 superagonist (IL-15 bound to the soluble IL-15Rα) effectively blocked HIV infection, in an NK cell dependent manner [66]. In parallel to these studies, multiple clinical trials of adoptive therapy with expanded allogeneic NK cells in cancer are assessing the therapeutic potential of ex vivo expansion and reinfusion into patients [67]. Results of these trials will be critical to define the safety of NK cell expansion and administration and may inform which subsets are critical to immune protection. Both of these approaches, in vivo exposure to IL-15 and ex vivo NK cell manipulation and reinfusion, have potential advantages, and further work is needed to define the most promising avenues.

Multiple additional pathways to modulate NK cell maturation and function are under investigation. Immunomodulatory therapies have potential efficacy in NK cells analogous to the effects described for the adaptive immune system; blocking PD-1 on NK cells from multiple myeloma patients enhanced killing of autologous tumor cells [68]. Indirectly, proteasome inhibitors used in cancer chemotherapy also enhance NK cell function, at least in part by downregulation of HLA molecules triggering missing self responses [69]. Due to their highly potent cytotoxic activity, an array of inhibitory receptors tonically repress the function of NK cells [44], offering another target for intervention. Direct blockade of inhibitory KIR receptors has been shown to augment spontaneous NK cell cytotoxicity in animal models [70] and is in exploratory clinical trials of cancer therapy [71].

### NK Cell Activation and Specificity

Efforts to modulate NK cells with cytokines and direct therapies point back to a critical question in NK cell biology. While there is considerable variation in NK cell response potential, the precise determinants (e.g., receptor expression and maturation state) of an NK cell response to pathogen challenge are not clear. In contrast to adaptive immune responses that are governed by antigen-specific gene-rearranged receptors, NK cells integrate multiple activating and inhibitory signals to reach a threshold of activation. Importantly, both the KIR genotype of an individual [22] and the multiple unique combinations of receptors that can be expressed [11, 12••] contribute to the NK population diversity of receptor expression and activation potential. However, apart from the genetic determinants, environmental and stochastic factors including infectious exposures such as CMV are also capable of profoundly shaping the response potential of NK cells through alterations in repertoire distribution [72, 73]. Building on differences in surface receptors, work defining transcriptional signatures of NK cells in distinct activation states has provided a context to understand NK cell activation alone and in relationship to innate lymphoid cells and traditional T cells [74]. Moving forward, to truly harness NK cells towards a therapeutic goal, the determinants of NK cell repertoire composition, activation threshold, and role in susceptibility to infection must be defined.

#### NK Cell Repertoire Diversity

Detailed studies with high dimensional analysis enabled by cytometry by time of flight (CyTOF) has provided novel insights into the diversity and specificity of the NK cell repertoire [11, 12••]. These studies reveal the potential for NK cell combinatorial receptor diversity, with estimates of up to 30,000 unique receptor phenotypes on a single NK cell in an individual subject. Remarkably, most individual receptor combinations contributed to only a minority of the overall NK cell population, with only the less mature NKG2A^+^/CD94^+^ NK cells and more mature CD57^+^/CD16^+^ population as primary distinct phenotypes [11]. Distinct responses to stimuli, proliferative potential, cytokine production, and cytolytic activity are seen in subsets defined by the expression of CD57 [75]. Diversity of phenotypes in adults is correlated with CD57 expression, suggesting that maturation leads to divergence, and was associated with risk of HIV acquisition. In case control analyses of high-risk women, increased NK cell diversity was associated with an increased probability of HIV-1 infection [12••]. It is not clear whether the NK diversity reflects a “distraction” from the challenging pathogen that increases susceptibility or if it is linked to other immune characteristics such as T cell exhaustion. These data raise questions about preventative and therapeutic vaccination goals; a naïve repertoire may have more flexibility and protective potential than one directed at other pathogens; however, a vaccine-directed, pathogen-specific response should be superior to both. Understanding how clonal NK cell populations can be reshaped and directed will critically inform the potential to harness a range of biological functions of NK cells through vaccine design.

#### NK Cell Memory

While classically considered as nonspecific innate effector cells, there is growing data to support adaptive, memory-like features of NK cells. These features can be divided into two categories, antigen-specific responses with classical recall characteristics and a more general alteration in effector function conditioned by an infection, vaccination, or inflammatory environment in line with the phenomenon of innate training in the myeloid compartment [14, 76]. Both types of memory responses are potential targets for immune intervention.

The mouse model of hapten-mediated contact hypersensitivity reactions provided the first demonstration of persistent, antigen-specific, and transferrable memory in NK cells [77]. Further work identified the importance of the chemokine receptor CXCR6 in memory development and extended the observations from haptens to influenza virus-like particles and inactivated vaccinia virus; the receptors mediating this recognition have remained undefined [78]. The first report of NK cell memory in the setting of a viral infection demonstrated NK cell-mediated responses to mouse cytomegalovirus (MCMV) [79]. Development of murine CMV (MCMV) memory was linked to interactions between the NK receptor LY49H and the viral glycoprotein m157 [80, 81], and memory cells were broadly distributed in the tissues, in contrast to the liver-resident hapten-directed memory cells [79]. More recently, memory NK cells specific for SIV antigens have been identified in the nonhuman primate model [13••]. NK cells derived from rhesus macaques infected with SIV or a virus combining SIV and HIV (SHIV) showed antigen-specific killing of targets pulsed with HIV protein components, and a persistent response to vaccine antigens was identified in animals who had received an adenovirus 26 (Ad26) vectored SIV vaccine 5 years earlier [13••]. These memory NK cells were elicited by both vaccination and infection, were durable and specific, and were recovered from both the liver and spleen [13••]. These animal model data demonstrate antigen-specific NK cell memory and while direct evidence in humans is lacking, these studies strongly support efforts to identify and harness this activity in human disease. Access to the relevant cell populations, e.g., hepatic or splenic cells, may be critical to identify these responses and should be one focus of study. In parallel, in vitro studies to induce memory-like response and animal models including the humanized mouse offer additional opportunities to explore the biology of these cytolytic effectors.

Antigen-specific memory in NK cells has clear parallels to traditional adaptive immunological memory. Consistent with their unique role bridging the innate and adaptive responses, NK cells are also capable of a memory phenotype that is induced by more generalized signals. Exposure of murine NK cells to cytokines (IL-12, IL-15, and IL-18) in vitro can induce a memory-like phenotype. When transferred into naïve hosts, these cells have a resting phenotype, but produce more IFNγ on cytokine restimulation, a response that remains detectable 3 weeks after cell transfer [82]. Human NK cells have similar properties, with a brief exposure to cytokines conditioning enhanced responsiveness on rechallenge [83]. How this memory-like state is established is a topic of investigation, but epigenetic remodeling of a noncoding sequence at the *IFNG* gene linked to increased IFN production can be induced by in vitro cytokine priming [84]. Epigenetic programming also underlies the sustained shifts in NK cell profiles that are seen in human CMV (HCMV) infection. HCMV infection drives expansion of a population of CD94-NKG2C NK cells [85], and in the setting of hematopoietic stem cell transplantation, this population has been demonstrated to have a memory-like response to CMV [86]. The interaction between HLA-E and CD94-NKG2C contributes to this expansion [87], but additional pathways to NK memory in HCMV are also operative, as evidenced by the FcεRIγ-deficient adaptive NK cells that expand after activation through CD16 [16••]. HCMV infection is associated with sustained changes in NK cell repertoire, distinct epigenetic profiles [15••, 16••], and altered functional profiles [15••, 16••]. The responses appear to be directed by exposure to the pathogen, in some cases directly through a viral antigen, and in other cases through secondary recognition of specific antibody. In congruence with the findings in HCMV, an NK cell population that lacks FcRγ expression and has enhanced ADCC activity was identified in HIV-infected subjects, with some features shared with the memory-like population induced by CMV and some unique surface receptor characteristics [88]. These findings have compelling links to both the biology of adaptive immune responses and the growing field of research in innate training and tolerance whereby epigenetic programs direct altered secondary responses after an initial exposure [14, 89], and both pathways can be harnessed towards goals of HIV prevention and cure.

### NK Cell Editing of Adaptive Immunity

Recent studies have focused attention on a critical role for NK cells in the shaping adaptive immune responses. In MCMV infection, NK cells rapidly eliminate infected targets, limiting the type I interferon response, preserving conventional dendritic cells and CD8 T cell responses [90]. In this infection, NK cells limit exposure of CD4 and CD8 T cells to infected dendritic cells shaping the subsequent adaptive response [91] and importantly, also limit tissue site T cell-mediated pathology [92]. In HIV, NK cell editing of dendritic cells is aberrant in the context of chronic inflammation and elevated IL-10, leading to poorly dendritic cells with limited immunogenicity [93]. Similarly, NK cells dictate immune response characteristics in an indirect fashion in the lymphocytic choriomeningitis virus (LCMV) mouse model. In this system, NK cells have no significant role in elimination of virus-infected targets, but they eliminate activated CD4 T cells either limiting immunopathology, or contributing to exhaustion and inefficient CD8 T cell control in chronic infection [94–96]. NK cells have also been shown to shape the induction of antibody; perforin-mediated elimination of T follicular helper (Tfh) cells in the lymph node by NK cells in acute infection was shown to disrupt germinal center formation, limiting immune memory development [97••]. Tfh cells have been identified as the dominant population supporting replication and virus production in viremic HIV-1 infection [98], and are likely a significant contributor to the HIV-1 reservoir.

The context-dependent effects of NK cells on adaptive immunity highlight the need for careful direction of efforts to harness their activity in HIV infection. Specifically, disabling their CD4 suppressive effects after vaccination may promote more breadth of antibody response. In contrast, during acute infection, enhancing NK-mediated elimination of Tfh cells may limit the size of the reservoir that is established. Likewise, during a curative intervention, unleashing NK cell targeting of Tfh cells could again lead to reservoir reduction. Directed recruitment of NK cells to lymph nodes also offers a pathway to increase their efficacy [99]. The impact of NK cells on interventions targeting adaptive responses offers a novel pathway to enhance efforts at prevention and cure [3].

## Conclusions

NK cells have emerged as multifunctional effector cells with the potential to control infections and shape adaptive immune responses. NK cells exert immune pressure on HIV and contribute to protective vaccine responses and some phenotypes of immune control. Significantly, there has been little effort to optimize, direct, or specifically target NK cells in therapeutic and preventative interventions. Mounting evidence from basic biological studies of NK cells points to the multiple avenues to enhance their antiviral and immunomodulatory function in HIV. The barrier to eliciting effective immunity to HIV is significant, and the potential to harness this important and potent effector population must be a high-priority component of research efforts in prevention and cure.
